# Quantitative electroencephalography as a next-generation tool in neurodiagnostics: significance, clinical applications, and practical interpretative frameworks

**DOI:** 10.3389/fnins.2026.1834391

**Published:** 2026-08-03

**Authors:** Marta Kopańska, Julia Trojniak, Maria Pachalska

**Affiliations:** 1 Department of Medical Psychology, Faculty of Medicine, University of Rzeszow, Rzeszów, Poland; 2 Student Research Club “Reh-Tech”, Faculty of Medicine, University of Rzeszów, Rzeszów, Poland; 3 Department of Neuropsychology and Neurorehabilitation, Andrzej Frycz Modrzewski Krakow University, Cracow, Poland

**Keywords:** differential diagnosis, Neurofeedback, neurophysiological biomarkers, precision psychiatry, QEEG, quantitative electroencephalography

## Abstract

**Introduction and objective:**

This article provides a comprehensive review of the methodology of Quantitative Electroencephalography (QEEG) as an advanced tool in modern neurodiagnostics. The objective of this study is to systematize knowledge regarding the technical aspects of signal acquisition, its specific clinical applications, and the interpretative frameworks that determine the efficacy of personalized therapeutic interventions in psychiatry and neurology.

**Materials and methods:**

This paper constitutes a critical review of the relevant literature. The study analyzed a total of 321 bibliographic sources, peer-reviewed empirical studies, systematic reviews, and supplementary scientific book chapters, published between 1932 and the first half of 2026.

**Results:**

The analysis of the gathered evidence demonstrates that QEEG has the potential to objectify the neurophysiological phenomena underlying clinical presentations. In neurodevelopmental disorders such as ADHD and ASD, this method may support the identification of electrophysiological subtypes and connectivity dysfunctions (e.g., the coexistence of hypo- and hypercoherence in autism). The technique has shown potential utility in differential diagnosis and the decomposition of overlapping symptoms. This includes unmasking hidden compensatory mechanisms in high-functioning patients with ADHD, which often manifest as hyperactivity in the Beta band. QEEG provides promising adjunctive biomarkers in affective disorders (such as Frontal Alpha Asymmetry - FAA) and anxiety disorders (characterized by an excess of fast waves). It is also being investigated as a potential adjunctive tool in detecting the early stages of neurodegeneration through a decrease in peak Alpha frequency and in exploring post-COVID syndromes. Furthermore, identifying individualized network profiles allows for the objective personalization of neuromodulatory therapies including Neurofeedback, rTMS, and tDCS. It also facilitates predicting and monitoring responses to pharmacotherapy, such as the innovative treatment of epilepsy with cannabidiol (CBD).

**Conclusion:**

QEEG is a promising translational tool that may help elevate neurodiagnostics from the level of subjective behavioral assessment to objective and measurable neurobiological indicators. Implementing this method in clinical practice serves as a valuable adjunctive tool to support diagnostic sensitivity and the development of personalized treatment strategies. It is hypothesized that this approach may potentially shorten the time required to achieve remission and minimize the risk of polypharmacy, though these clinical benefits require further validation through prospective controlled studies.

## 1 Introduction

Modern medicine, particularly disciplines dealing with the examination and treatment of the central nervous system (CNS) such as neuropsychology, psychiatry, and neurology, is undergoing a dynamic technological transformation. An evolution of the diagnostic paradigm is observed, which involves shifting away from an exclusive reliance on the subjective assessment of clinical symptoms toward the identification of objective and measurable neurophysiological biomarkers (Lleó, 2021; Abi-Dargham et al., 2023). In this new approach, Quantitative Electroencephalography (QEEG), commonly referred to as “brain mapping,” emerges as a tool of growing clinical relevance. It integrates traditional psychiatric observation with precise analytics (Popa et al., 2020; Yao et al., 2022). The role of QEEG is systematically growing as it offers detailed insights into the functional architecture of neuronal networks that remain beyond the reach of standard structural neuroimaging methods such as magnetic resonance imaging (MRI) or computed tomography (CT) (Popa et al., 2020).

While conventional electroencephalography (EEG), originating from the pioneering late 19th and early 20th-century works of Caton, Beck, and Berger, established the foundation of clinical neurophysiology, it historically relied almost exclusively on qualitative visual inspection (Coenen and Zayachkivska, 2013; Rossini et al., 2025). Although highly sensitive for detecting gross paroxysmal pathologies, classical EEG is inherently limited in its ability to objectively identify subtle, network-level dysfunctions (Duffy et al., 1979; Wallace et al., 2001; Misciagna, 2021). To overcome these limitations, the necessity to objectify bioelectrical data led to the implementation of QEEG, operationalizing the raw signal through digital processing and topographic mapping.

The necessity to objectify data led to the implementation of quantitative analysis. Its mathematical principles were formulated in 1932 by G. Dietsch, but full operationalization only became possible in the era of digital signal processing and the development of topographic mapping techniques (BEAM) by F. Duffy in the 1980s (Dietsch, 1932; Duffy et al., 1979; Bronzino, 1984; Popa et al., 2020). The core of QEEG methodology currently relies on the application of the Fast Fourier Transform (FFT) algorithm (Zhang et al., 2023). This procedure enables the conversion of the raw EEG signal from the time domain to the frequency domain, generating a precise power spectrum. This allows for the decomposition of the complex signal into orthogonal frequency bands (Delta, Theta, Alpha, Beta, Gamma) and the estimation of network indicators such as phase coherence and interhemispheric asymmetry. According to research on the pathophysiology of Autism Spectrum Disorder (ASD), such focused analysis enables the detection of CNS microdysfunctions that remain undetectable during traditional curve evaluation (Kopańska et al., 2025c).

The significant increase in QEEG implementation in clinical practice is a direct response to psychiatry’s demand for precision medicine solutions. It provides specific biomarkers where behavioral categorizations based on DSM or ICD prove insufficient. For instance, in evaluating patients with Attention-Deficit/Hyperactivity Disorder (ADHD), quantitative analysis reveals highly reproducible patterns involving increased spectral density in the Theta band combined with a deficit in Beta activity. This supports the Theta/Beta ratio (TBR) as an adjunctive diagnostic marker (McVoy et al., 2019a). This methodology also demonstrates potential utility in the long-term monitoring of the effectiveness of innovative interventions, such as the use of cannabidiol (CBD) in drug-resistant epilepsy, where changes in spectral parameters precede measurable clinical improvement (Kopańska et al., 2025d).

Analyzing the potential of QEEG also requires reference to neuromodulation based on biological feedback known as Neurofeedback. These interventions essentially represent the clinical translation of data acquired through quantitative analysis. While QEEG examination serves to identify localized dysregulations, Neurofeedback enables the targeted correction of these aberrations through precisely applied operant conditioning paradigms and the stimulation of neuroplasticity (Kamiya, 1968; Sterman et al., 1969; Barry Sterman, 1996). Clinical studies involving pediatric patients with mild autism spectrum symptoms have suggested that training protocols personalized based on the baseline QEEG profile may correlate with improvements in the domains of executive functions and attention control (Kopańska et al., 2025c).

Currently, QEEG positions itself as a highly interdisciplinary research tool. Its applicability extends beyond traditional uses to include the diagnosis of complex phenomena. A notable example is the diagnosis of ADHD in high-functioning adults, where compensatory mechanisms mask primary deficits (Kopańska and Trojniak, 2025). Concurrently, a growing body of literature indicates the utility of QEEG in objectifying affective and anxiety disorders and in exploring the neurophysiological correlates of chronic neuropathic pain (Chmiel et al., 2026).

The aim of this article is to provide a critical review of QEEG methodology as an advanced tool in modern neurodiagnostics. The subsequent sections of this study provide a detailed analysis of the technical aspects of the technique, its specific nosological implementations, and the interpretative frameworks that determine the effective translation of electrophysiological data into precise therapeutic strategies in clinical settings.

## 2 Literature search strategy and selection criteria

To provide a comprehensive and clinically oriented overview of the current evidence of the QEEG landscape, a narrative review methodology was employed. A structured literature search was conducted across major electronic databases, including PubMed/MEDLINE, Scopus, and Web of Science. The final narrative review included 321 bibliographic sources published between 1932 and the first half of 2026. The search strategy combined controlled vocabulary and free-text keywords related to QEEG methodology and its clinical applications. Representative search terms included: (“Quantitative Electroencephalography” OR “QEEG” OR “EEG biomarkers”) AND (“neurofeedback” OR “neuromodulation” OR “ADHD” OR “depression” OR “Alzheimer’s” OR “pain”).

Inclusion criteria comprised peer-reviewed journal articles, systematic reviews, meta-analyses, and official clinical guidelines published in English. To provide historical context, seminal publications describing the development of QEEG methodology were also included alongside contemporary studies. Conversely, studies with insufficient methodological reporting to allow critical appraisal of the presented findings and isolated case reports were generally excluded unless they described historically important methodological developments or unique clinical observations relevant to the evolution of QEEG. The final literature selection prioritized high-quality Randomized Controlled Trials (RCTs) and consensus statements to adequately stratify the level of evidence across various clinical applications.

While scientific book chapters were included for historical and conceptual background, all major clinical claims and efficacy assessments were rigorously derived exclusively from peer-reviewed empirical studies and systematic reviews.

To reduce selection bias inherent to narrative reviews, evidence was intentionally synthesized from multiple databases, priority was given to evidence replicated across independent research groups, systematic reviews, consensus statements, and high-quality prospective studies, while findings derived primarily from isolated case reports or single research groups were interpreted cautiously or single research groups were interpreted cautiously and presented within the context of the broader literature.

As this manuscript was designed as a narrative review rather than a systematic review, no formal PRISMA-based study flow diagram or predefined sequential screening process was applied. Accordingly, the objective was to synthesize and critically discuss the current state of knowledge rather than to provide an exhaustive quantitative evidence synthesis. Instead, the literature search served to representative publications covering the major methodological developments and clinical applications of QEEG, with preference given to studies providing higher levels of evidence. Given the narrative nature of this review, no formal study quality appraisal tool (e.g., AMSTAR-2, CASP, or JBI critical appraisal checklists) was applied. Instead, greater weight was given to higher levels of evidence, including clinical guidelines, meta-analyses, systematic reviews, randomized controlled trials, and large prospective studies whenever available.

## 3 Methodology and architecture of QEEG examination: from signal acquisition to spectral analysis

### 3.1 Technological foundations and spectrum analysis

QEEG methodology operationalizes the raw electroencephalographic signal as a substrate for advanced, quantitative spectral analysis. The central algorithm of this process is the FFT, which determines the mathematical conversion of the recording from the time domain to the frequency domain (Kropotov, 2009).

To achieve this, computations in clinical neurophysiology are performed exclusively utilizing optimized FFT algorithms. This optimization drastically reduces computational complexity, constituting a technological prerequisite for the seamless generation of topographic brain maps and the execution of spectral analyses in real time without analytical latency (Kropotov, 2009).

This procedure enables the precise estimation of Power Spectral Density (PSD), allowing for the calculation of absolute and relative signal power parameters (Kopańska et al., 2024). The decomposition of the output signal results in its division into orthogonal, classic frequency bands: Delta, Theta, Alpha, Beta, and Gamma, the standard ranges of which are presented in Table 1.

**Table 1:** Adult wave frequency range developed by Kopańska et al. (2025b).

Wave	Range (Hz)
Delta	0.5–3
Theta	4–8
Alpha	8–12
SMR	12–15
Beta	15–20
Beta2	20–34

Standard QEEG protocols require digital recording, specific filters (e.g., 0.5–45 Hz, notch 50/60 Hz), division into epochs (1–2 s), FFT with an appropriate window (e.g., Hanning), and spectrum averaging (Bellato et al., 2020; Chang and Chang, 2023; Ortega-Leonard and del Río-Portilla, 2023). Data prepared in this manner form numerical matrices that serve as the foundation for statistical analyses, band ratios (e.g., Theta/Alpha, Theta/Beta), and clinical biomarkers (Bellato et al., 2020; Chang and Chang, 2023; Ghuli et al., 2024). However, it is crucial to acknowledge that QEEG outcomes are highly sensitive to methodological heterogeneity (Robbins et al., 2020; Keizer, 2021; Troller-Renfree et al., 2025). Variations in analytical parameters, such as the choice of window functions (e.g., Hanning vs. Hamming), epoch lengths, and trace processing strategies, can yield significantly divergent power spectral density estimates (Levy, 1987; Alam et al., 2020; Ahuis et al., 2024). Consequently, maintaining strictly standardized analytical pipelines is imperative to ensure clinical reliability (Keizer, 2021; Ahuis et al., 2024; Collura et al., 2025).

While standard FFT provides robust spectral power estimates, it inherently assumes signal stationarity and primarily captures linear dynamics (Thakor and Tong, 2004; Ma et al., 2018; Arts and van den Broek, 2022). To address the complex, non-stationary nature of neural oscillations, modern core QEEG analysis has expanded to include advanced mathematical frameworks (Thakor and Tong, 2004; Popa et al., 2020). The continuous wavelet transform (CWT) is increasingly utilized to resolve the time-frequency trade-off, making it highly effective for capturing transient EEG events and non-stationary signals that standard FFT might obscure (Thakor and Tong, 2004; Bozhokin and Suslova, 2015; Arts and van den Broek, 2022). Furthermore, acknowledging the brain as a complex chaotic system, nonlinear dynamics methods, such as entropy and complexity measures, are now frequently employed (Thakor and Tong, 2004; Ma et al., 2018; Lau et al., 2022). These parameters quantify the unpredictability and irregularity of the EEG signal, offering sensitive biomarkers for subtle neurophysiological shifts (Lau et al., 2022; Yuan and Zhao, 2025). Additionally, the temporal domain analysis has been enriched by the study of EEG microstates, transient, topographically stable global brain states lasting tens of milliseconds (Khanna et al., 2015; Keller et al., 2023; Yuan and Zhao, 2025). Often conceptualized as the “atoms of thought,” microstates provide a direct, high-resolution electrophysiological window into the temporal dynamics of large-scale resting-state functional networks (Van De Ville et al., 2010; Michel and Koenig, 2018; Nagabhushan Kalburgi et al., 2024).

Beyond resting-state baseline parameters, the combined utilization of continuous QEEG and Event-Related Potentials (ERPs) represents a vital complement in contemporary clinical diagnostics (Popa et al., 2020; Cecchi et al., 2023; Young et al., 2025). While QEEG delineates the tonic functional architecture and baseline spectral properties of neuronal networks, ERPs provide a dynamic measure of brain reactivity time-locked to specific sensory, cognitive, or motor stimuli (Popa et al., 2020; Paitel et al., 2021; Liang et al., 2025). Integrating resting-state QEEG profiles with the assessment of specific ERP components, such as the P300 wave, which indexes directed attention and context updating, may support a comprehensive evaluation of neurocognitive integrity (Donchin and Coles, 1988; Kaiser et al., 2020; Ganapathi et al., 2022; Cecchi et al., 2023; Young et al., 2025). This combined methodology is of great clinical interest, as it bridges the gap between resting brain states and active, phasic information processing, thereby providing a more comprehensive functional assessment in conditions such as neurodevelopmental disorders and cognitive decline (Kaiser et al., 2020; Ganapathi et al., 2022; Young et al., 2025).

It is also crucial to emphasize that the current landscape of QEEG data analysis is fundamentally driven by advancements in machine learning (Saeidi et al., 2021; Gkintoni et al., 2025; Uyanik et al., 2025). Today, artificial neural networks (ANNs), particularly deep learning architectures such as Convolutional Neural Networks (CNNs) and Recurrent Neural Networks (RNNs), have emerged as the most important and powerful methods for extracting and analyzing relevant EEG features (Craik et al., 2019; Roy et al., 2019; Parsa et al., 2023). Unlike traditional analytical pipelines that rely heavily on manual feature engineering, neural networks can automatically learn and identify complex, non-linear spatiotemporal patterns directly from high-dimensional raw or preprocessed EEG data (Roy et al., 2019; Rivera et al., 2022). This advanced feature extraction capability not only optimizes the automated classification of specific neuropsychiatric disorders but also significantly enhances the detection of subtle, multidimensional neurophysiological biomarkers that conventional spectral analyses might inherently overlook (Lawhern et al., 2018; Parsa et al., 2023; Yun, 2024).

### 3.2 Acquisition and the 10–20 system

**Figure 1:**
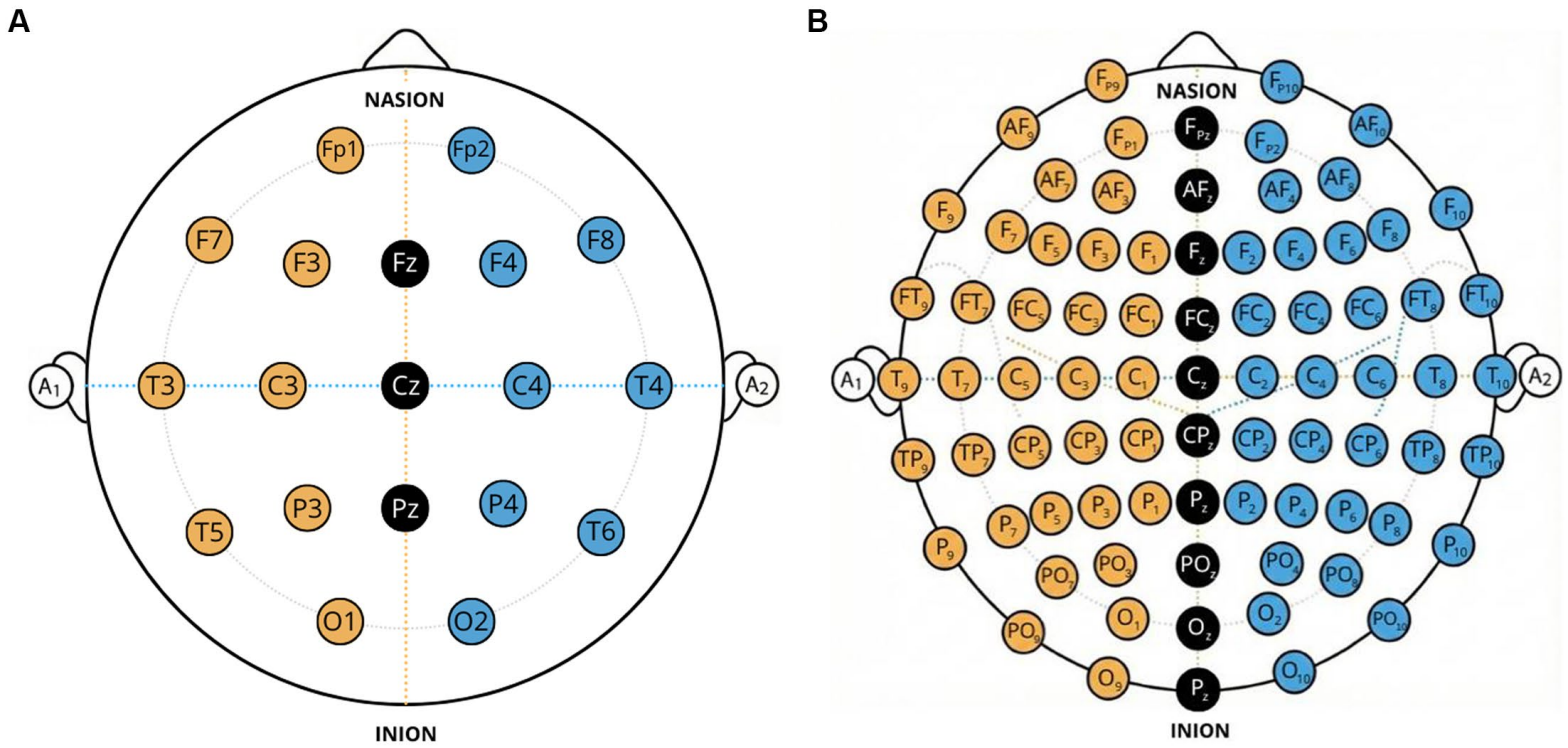
Comparison of electrode layouts in the 10–20 **(A)** and 10–10 **(B)** systems.

The foundation of reproducible and methodologically rigorous electroencephalographic data acquisition is the standardization of the spatial distribution of measurement sensors. The classic 10–20 system, originally developed in 1958 by Jasper, relies on proportional distances (10 and 20%) between fixed anatomical craniometric points including the nasion, inion, and bilateral preauricular points—(Figure 1; Jasper, 1958; Klem et al., 1999). Although this system unified global clinical neurophysiology, the development of QEEG and the demand for high-resolution spatial mapping forced the implementation of its denser extensions. These include the 10–10 system introducing 74 standard positions and the 10–5 system allowing for the application of approximately 345 electrodes (Oostenveld and Praamstra, 2001).

The necessity of employing denser measurement matrices (High-Resolution EEG, HREEG) stems directly from the biophysical limitations of electrical signal propagation. Potentials generated by cortical dipoles undergo strong attenuation and spatial dispersion when passing through layers of varying electrical resistance, including cerebrospinal fluid, meninges, the skull, and the scalp. This phenomenon is defined as volume conduction. The application of 10–10 and 10–5 systems fulfills the requirements of the spatial Nyquist theorem. This drastically reduces the phenomenon of spatial aliasing and enables the precise separation of closely adjacent cortical signal sources (Robinson et al., 2017; Seeck et al., 2017; Heine et al., 2020).

A key argument for utilizing expanded topographic systems is their highly stable correlation with the underlying neuroanatomical structures. Modern research utilizing multimodal MRI-EEG coregistration demonstrates that standardized electrode positions in the 10–10 system exhibit rigorous constancy relative to specific gyri and sulci of the cerebral cortex, regardless of individual differences in skull morphology (Jurcak et al., 2007; Tsuzuki et al., 2017). The use of neurosurgical navigation systems and digital digitization of electrode positions relative to fiducial markers has shown that these positions can be localized relative to the cerebral cortex with a spatial deviation not exceeding a few millimeters (Koessler et al., 2009). Such precision allows for the direct mapping of observed spectral changes to specific Brodmann areas (Jurcak et al., 2007; Koessler et al., 2009). This extension is currently the official standard of the American Clinical Neurophysiology Society and the International Federation of Clinical Neurophysiology (IFCN) and forms the basis of modern high-density nets (Seeck et al., 2017).

However, the clinical implementation of high-density EEG (HD-EEG) arrays, such as the 10–10 or 10–5 systems, is accompanied by significant practical challenges (Stoyell et al., 2021; Fiedler et al., 2022). The application of dense montages substantially increases patient preparation time, operational complexity, and overall equipment costs (Tenke and Kayser, 2001; Mumtaz et al., 2021; Stoyell et al., 2021; Fiedler et al., 2022; Runstadler et al., 2025). Furthermore, the close proximity of electrodes in HD-EEG setups elevates the risk of saline or conductive gel bridging, leading to cross-channel signal contamination and a higher overall artifact burden that complicates preprocessing pipelines (Tenke and Kayser, 2001; Mumtaz et al., 2021).

Another critical technical consideration is hardware quality, particularly in light of the growing market for low-cost, consumer-grade EEG devices (Minguillon et al., 2017; Amico and Koberda, 2025). Many of these commercial systems operate at low sampling frequencies (e.g., below 250 Hz). A high and stable sampling rate is a fundamental prerequisite for robust QEEG analysis. Inadequate sampling not only precludes the accurate evaluation of fast frequency networks (such as high-Beta and Gamma bands) but also introduces the risk of temporal aliasing, which fundamentally degrades the reliability of the derived clinical biomarkers (Crone et al., 2006; Gliske et al., 2016; Lundy et al., 2023; Jamil et al., 2024; Clayson, 2025; Collura et al., 2025).

Equally crucial to the spatial configuration of active sensors is the selection of an appropriate reference scheme, as this choice profoundly impacts the resulting QEEG topography, absolute power estimates, and functional connectivity metrics (Chella et al., 2016; Yang et al., 2017; Dong et al., 2023). While physical references, such as linked earlobes or mastoid processes, are historically prevalent in clinical settings, they are not electrically neutral and can introduce systematic bias, particularly affecting the amplitude of adjacent temporal regions (Chella et al., 2016; Yang et al., 2017). Conversely, computational references, such as the common average reference (CAR) or the Reference Electrode Standardization Technique (REST), aim to approximate a zero-potential point (Lei and Liao, 2017; Yang et al., 2017; Dong et al., 2023). It must be noted, however, that CAR requires a high-density, evenly distributed electrode array over the entire head to be mathematically valid (Chella et al., 2016; Lei and Liao, 2017; Candia-Rivera et al., 2021). Otherwise, it may generate spurious topographies (Yang et al., 2017). Consequently, the interpretative framework of QEEG must rigorously account for the applied reference, as different schemes can yield significantly divergent amplitude maps and coherence values (Chella et al., 2016; Lei and Liao, 2017).

### 3.3 Resting protocol EC/EO and alpha reactivity

Resting state EEG is typically recorded in eyes-closed (EC) and eyes-open (EO) blocks, lasting from several dozen seconds to a few minutes for each condition (Babiloni et al., 2024). Under eyes-closed conditions, it is possible to assess the brain’s basic resting state. In this state, the Alpha rhythm (8–12 Hz) dominates in the occipito-parietal regions, which is considered the canonical indicator of the visual cortex readiness state (Wan et al., 2019; Han et al., 2022).

Opening the eyes leads to physiological Alpha desynchronization (the so-called blocking response) and a distinct decrease in power within this band, especially in posterior regions (Wan et al., 2019; Babiloni et al., 2024; Ingram et al., 2024; Krukow et al., 2024). Source analyses (sLORETA, eLORETA) reveal that the state change from EC to EO involves not only the occipital cortex but also global attention and executive networks (Babiloni et al., 2022, 2024; Krukow et al., 2024).

In a healthy population, a strong decrease in Alpha power (over 10% reduction) is observed during the transition from the EC to EO state in the occipito-parietal cortex of most subjects, including the elderly (Babiloni et al., 2022, 2024). Concurrently, functional connectivity in posterior networks decreases in the Alpha band. The desynchronization after opening the eyes is rapid (≤1 s), while the return of synchronization upon closing the eyes is slower, suggesting different dynamics of network reorganization for the EC to EO and EO to EC transitions (Krukow et al., 2024).

Research therefore indicates that the magnitude of this Alpha reactivity (EC-EO) is linked to the efficiency of the cholinergic system and overall cognitive health. Weakened Alpha desynchronization upon eye opening is associated with attention deficits and poorer cognitive functioning in conditions such as ADHD, learning disorders, postoperative attention disorders, and dementias (Wan et al., 2019; Clarke et al., 2020; Acker et al., 2024; Kumru, 2025).

### 3.4 Apparatus and examination procedure

A standard QEEG examination is typically performed using an electroencephalographic cap compliant with the international 10–20 system. This ensures reproducible anatomical relations between the electrodes and the underlying cortical areas, which has been confirmed in studies of electrode projections and cranio-cerebral correlations. In clinical and research practice, a 19-channel montage is most frequently used, encompassing the standard set of 10–20 electrodes along with reference and ground electrodes usually placed on the earlobes or mastoid processes (Del Percio et al., 2025; Simfukwe et al., 2025a). The quality of the recording depends crucially on the parameters of the skin-electrode contact. Most clinical protocols require an impedance below 5 kilohms, which limits noise and improves the signal-to-noise ratio (Schumacher et al., 2020; Simfukwe et al., 2025a). It has been shown that higher impedance favors the increase of low-frequency noise and may force a larger number of trials to obtain stable power spectrum estimates, especially in less controlled environmental conditions (Kappenman and Luck, 2010; Mathewson et al., 2017).

Another critical element is the control of muscle artifacts. Methodological reviews and spectrum analyses indicate that EMG activity from the forehead, jaw, and neck muscles strongly contaminates the Beta and Gamma bands. It often exceeds the amplitude of the cortical signal and leads to a false inflation of high-frequency power (Lejko et al., 2020). Therefore, the importance of careful skin preparation, stable electrode placement, and instructions limiting movement, blinking, jaw clenching, or swallowing is strongly emphasized (Paitel et al., 2025; Simfukwe et al., 2025a). In children, where muscle tension and anxiety are more frequent, additional anxiety reduction strategies are recommended. These include explaining the examination process, gradual habituation, and the presence of a caregiver. This significantly increases the proportion of artifact-free segments and improves the reliability of analyses in the Beta and Gamma bands (Lejko et al., 2020).

Resting QEEG recording is typically conducted in two conditions: with eyes open (EO) and closed (EC). The registration of each position lasts at least several dozen seconds to a few minutes, from which a total of ≥60–90s of pure, artifact-free signal is selected (Edgar et al., 2023; Zawiślak-Fornagiel et al., 2024; de Jonge et al., 2025). Each of these states activates different neuronal networks. Such a two-state protocol allows the assessment not only of the power distribution in classic frequency bands but also of Alpha rhythm reactivity. In numerous studies, this reactivity has proven to be a sensitive indicator of visual-attentional network integrity, cholinergic system efficiency, and cognitive condition in both children and elderly individuals with neurodegenerative disorders (Acker et al., 2024; Zawiślak-Fornagiel et al., 2024; de Jonge et al., 2025).

Furthermore, the resting-state EEG is exquisitely sensitive to momentary fluctuations in patient vigilance (Del Percio et al., 2017; Falahpour et al., 2018; Chen et al., 2020). It is crucial to monitor the arousal state during data acquisition, as transient drowsiness can induce a physiological dropout of the Alpha rhythm and a concomitant increase in Theta activity (Babiloni et al., 2025; Salamone et al., 2025; Carpi et al., 2026). Without careful clinical observation, these vigilance-related shifts risk being erroneously interpreted as pathological cortical slowing characteristic of neurodegeneration or attention deficits (Østergaard et al., 2024; Salamone et al., 2025).

### 3.5 Data analysis and artifacts

The raw EEG signal has a very low amplitude and is therefore extremely sensitive to disturbances. Artifacts can originate from biological sources such as blinking, eye movements, jaw and neck muscle activity, or the ECG signal. They can also arise from technical sources including 50/60 Hz power line noise, high electrode impedance, or equipment drifts (Jung et al., 2000; Mannan et al., 2018; Jiang et al., 2019). Before proceeding with quantitative analysis, the recording must undergo a meticulous preprocessing procedure involving the detection and correction or rejection of artifacts.

Modern EEG packages use advanced blind source separation methods, primarily Independent Component Analysis (ICA). Assuming a linear mixing of signals, this method allows for the mathematical separation of brain components from artifacts without the necessity of recording additional reference channels (Jung et al., 2000; Mannan et al., 2018; Artoni and Michel, 2025). Once the components corresponding to artifacts are identified, they are rejected, and the EEG signal is reconstructed exclusively from neuronal components (Jung et al., 2000; Jiang et al., 2019; Pedroni et al., 2019; Artoni and Michel, 2025). Increasingly, ICA is combined with other approaches such as bandpass filtering, wavelets, PCA, and automatic IC classification. This allows the automation of the process and limits the risk of losing essential brain data (Mannan et al., 2018; Jiang et al., 2019; Pedroni et al., 2019; Bailey et al., 2023; Arpaia et al., 2025). Only such a “cleaned” recording, usually comprising at least several dozen seconds to a few minutes of relatively artifact-free signal for each condition depending on the paradigm and analysis requirements, is subjected to proper statistical analysis (Kopańska et al., 2025b).

Despite its widespread adoption, Independent Component Analysis (ICA) is not without significant methodological limitations (Pion-Tonachini et al., 2019; Kim et al., 2023). The algorithm inherently assumes that signal sources are statistically independent and spatially stationary, which may not always align with the highly dynamic and non-stationary nature of neural generators (Hsu et al., 2016, 2018). Furthermore, there is a persistent risk of “signal leakage,” where true neural activity is inadvertently removed along with artefactual components, or conversely, residual artifacts are left intact (Issa and Juhasz, 2019; Dimigen, 2020). The manual classification of independent components also introduces a degree of subjective bias, although the increasing use of automated classification algorithms aims to mitigate this issue (Radüntz et al., 2017; Pion-Tonachini et al., 2019). Finally, the efficacy of ICA is heavily dependent on the number of recording channels and the length of the continuous data, meaning its performance and reliability can degrade significantly in low-density or short-duration EEG recordings (Hsu et al., 2016; Maddirala and Veluvolu, 2022).

### 3.6 Normative databases and Z-scores: the essence of QEEG diagnosis

What distinguishes QEEG from classic EEG is the statistical comparison of patient parameters with normative databases based on large samples of healthy individuals, divided by age and sex (Ko et al., 2021). This allows for the transformation of raw wave power values (microvolts squared, μV^2^) into Z-scores, which describe the deviation of a given parameter from the population mean in units of standard deviation (Thatcher et al., 2003; Collura, 2020; Ko et al., 2021).

QEEG databases typically assume a distribution close to normal (mean 0, SD = 1), allowing the interpretation of the ±1.96 SD range as covering approximately 95% of the population (Thatcher et al., 2003; Ko et al., 2021). Values falling outside this interval indicate a statistically significant deviation and can be treated as a marker of brain activity dysregulation (Thatcher et al., 2003; Collura, 2020; Ko et al., 2021; Wu and Lin, 2023). Z-scores can be calculated for absolute power, relative power, asymmetry, coherence, and phase (Collura, 2020; Jeong et al., 2022; Wu and Lin, 2023).

However, a critical limitation in the application of Z-scores is the inherent variability and potential bias within normative databases (Prichep, 2005; Wood et al., 2024). Different commercially available databases utilize distinct inclusion criteria, preprocessing pipelines, and hardware setups, which can lead to inter-database discrepancies in the output Z-scores (Lorensen and Dickson, 2003; Young et al., 2024). More importantly, diagnostic bias can emerge from inadequate demographic stratification (Johnstone and Gunkelman, 2003; Young et al., 2024). Many databases are built on samples that may lack sufficient diversity regarding race, ethnicity, and genetic backgrounds (Johnstone and Gunkelman, 2003; Morales et al., 2025). Since baseline EEG spectral parameters can be subtly influenced by these demographic variables, applying a homogenous normative database to a diverse clinical population carries the risk of misinterpreting benign physiological variants as pathological deviations (Smit et al., 2005; Hernandez et al., 2024). Therefore, robust and granular age stratification, alongside diverse, representative sampling, is essential to ensure the cross-cultural validity of QEEG diagnostics (Prichep, 2005; Bosch-Bayard et al., 2020; Miranda et al., 2021).

From a statistical perspective, generating comprehensive topographic maps involves simultaneously analyzing variables across dozens of channels, frequency bands, and functional connectivity metrics (Vialatte and Cichocki, 2008; Miranda et al., 2021). This high-dimensional analysis drastically inflates the risk of Type I errors (false positives) (Hemmelmann et al., 2005; Vialatte and Cichocki, 2008; Wood et al., 2024). Consequently, without applying stringent statistical corrections for multiple comparisons, there is a substantial risk of overinterpreting random signal variations as clinically meaningful biomarkers (Vialatte and Cichocki, 2008; Wood et al., 2024). Finally, the test–retest reliability of these QEEG parameters can vary significantly depending on the specific metric and recording conditions, underscoring the absolute necessity for rigorous, standardized acquisition protocols to ensure clinical consistency across repeated evaluations (Roberts et al., 2016; Keizer, 2021; Collura et al., 2025).

The application of Z-scores in QEEG allows for the detection of patterns characteristic of specific disorders, such as changes in band power, Frontal Alpha Asymmetry (FAA) in depression, or abnormal connectivity in dementia (Popa et al., 2020; Ko et al., 2021; Simfukwe et al., 2023, 2025b; Yuan and Zhao, 2025). In patients with neuropsychiatric disorders, deviations in the Theta/Alpha bands and in coherence and phase parameters are frequently observed despite a normal classic EEG (Simfukwe et al., 2023; Kopańska et al., 2025e).

Multidimensional neurophysiological assessment in the QEEG paradigm goes beyond local spectral power analysis by including the advanced estimation of functional connectivity parameters. These indicators objectify the communication efficiency and coupling stability within distributed neuronal networks. Key estimators used in clinical diagnostics are presented in Table 2.

**Table 2:** Functional connectivity estimators in QEEG and their clinical validation.

Network parameter	Electrophysiological definition and characteristics	Clinical significance and objectified biomarkers	Sources
Coherence	Quantification of linear synchronization and amplitude-phase agreement of the signal between topographically defined cortical areas.	Pathologically reduced coherence (hypocoherence) in the Theta, Alpha, and Beta bands constitutes an established marker of disconnection syndromes, including mild cognitive impairment (MCI) and dementia.	Popa et al. (2020), Simfukwe et al. (2023), (2025b), and Zawiślak-Fornagiel et al. (2024)
Phase	Estimation of the stability of nonlinear phase differences and the directionality of signal transmission. Indicators such as the Phase Lag Index (PLI) exhibit significantly higher resilience to volume conduction artifacts compared to classic coherence.	Evaluation of transmission pathway integrity. Phase indicators allow for the exclusion of common sources’ impact, lending credibility to the analysis of network dynamics.	Stam et al. (2007), Zawiślak-Fornagiel et al. (2023), Simfukwe et al. (2025b), and Yuan and Zhao (2025)
Interhemispheric asymmetry	Statistical analysis of differences in power spectral density (PSD) between topographically homologous areas of the right and left brain hemispheres.	FAA is currently investigated as an adjunctive indicator of predisposition to affective disorders, including depressive episodes, though it should not be used as a standalone diagnostic criterion due to outcome heterogeneity.	Popa et al. (2020) and Kopańska et al. (2024)

### 3.7 Advantages and limitations of the method

An objective evaluation of the clinical utility of the QEEG method requires a critical balancing of its diagnostic properties. The primary advantage of this technique is its high temporal resolution on the order of milliseconds, outclassing hemodynamic and metabolic neuroimaging such as fMRI or PET (de las Heras et al., 2024). This property enables the precise monitoring of the sub-second dynamics of neuronal processes in real time (Fingelkurts and Fingelkurts, 2022). Furthermore, QEEG is characterized by a high safety profile, cost-effectiveness, and the possibility of repeated serial measurements at the patient’s bedside. This makes the technique particularly useful in the long-term monitoring of therapies (de las Heras et al., 2024).

The critical limitation of the technique remains its low spatial resolution. The signal recorded from the scalp surface represents the spatiotemporal summation of the activity of millions of neurons, which undergoes significant attenuation and spatial dispersion during propagation through media of varying impedance, including cerebrospinal fluid, meninges, and skull bone structures. This phenomenon, defined as volume conduction, constitutes the main source of topographic distortions (Asadzadeh et al., 2020; Frühwirt et al., 2025). To partially mitigate the profound issue of volume conduction, advanced source localization algorithms, such as Standardized Low-Resolution Brain Electromagnetic Tomography (sLORETA) and Exact LORETA (eLORETA), have been integrated into modern QEEG analyses (Asadzadeh et al., 2020; Dattola et al., 2020). These algorithms mathematically estimate the three-dimensional distribution of intracortical electrical generators, effectively reducing scalp-level blurring and allowing for a more accurate topological localization of network hubs (Cincotti et al., 2004; Pascual-Marqui et al., 2011; Rao and Sailaja, 2024). However, it must be objectively acknowledged that the neuroanatomical precision of even the most advanced EEG source imaging fundamentally lags behind that of functional Magnetic Resonance Imaging (fMRI) (Asadzadeh et al., 2020; Tejay and Mohammed, 2023; Zeltser et al., 2024; Collura et al., 2025; Frühwirt et al., 2025). Because the EEG inverse problem is mathematically ill-posed, as an infinite number of internal source configurations could theoretically produce the same scalp topography, LORETA methods provide a statistically probable estimation rather than a direct structural measurement (Pascual-Marqui, 2002; Dattola et al., 2020). Consequently, they yield a spatial resolution on the order of centimeters, which cannot compete with the millimeter precision offered by fMRI (Asadzadeh et al., 2020; Dattola et al., 2020; Zeltser et al., 2024).

An additional analytical challenge is the high competency threshold required for result interpretation. Automated statistical reports generated directly by software carry the risk of overinterpretation without rigorous verification by an experienced clinician. Proper data validation requires advanced neurophysiological knowledge that determines the accurate differentiation of genuine pathological aberrations from physiological normal variants and subtle non-biological artifacts (Popa et al., 2020; de las Heras et al., 2024).

## 4 Clinical applications and new diagnostic horizons of QEEG: from neurodevelopmental disorders to pain medicine

The application of QEEG systematically expands the capabilities of modern neurodiagnostics. This method enables the transition from the analysis of macroscopic structural damage to the objective assessment of disorders at the level of dynamics and functional connectivity of neuronal networks. This contributes to the development of precision psychiatry by allowing the identification of electrophysiological indicators underlying specific clinical symptoms.

To contextualize the varied diagnostic utility of QEEG, Table 3 summarizes the current evidence base, representative findings, and clinical readiness across different neuropsychiatric conditions.

**Table 3:** Summary of QEEG clinical applications, evidence levels^1^, limitations and clinical readiness.

Clinical application	Representative QEEG findings	Level of evidence^1^	Major limitations	Current clinical status
Attention-deficit/hyperactivity disorder (ADHD)	Elevated TBR frontocentrally; compensatory excess of high-Beta activity in high-functioning adults.	Moderate	Significant developmental variability; findings often overlap with anxiety, fatigue, or sleep deprivation.	Adjunctive assessment (FDA-cleared for TBR)
Autism spectrum disorder (ASD)	Atypical connectivity architecture (coexistence of local hypercoherence and long-range hypocoherence).	Moderate	Extreme clinical heterogeneity; high risk of motor and myogenic artifacts during data acquisition in pediatric populations.	Research use/Adjunctive assessment
Major depressive disorder (MDD)	FAA (left-sided hypoactivation/increased Alpha power relative to the right hemisphere).	Moderate	Small and variable effect sizes; high outcome heterogeneity across studies; trait vs. state dependency.	Research use/Adjunctive assessment
Anxiety disorders	Generalized or localized excessive High-Beta (20–30 Hz) power reflecting chronic cortical hyperarousal.	Low to moderate	High-frequency bands are severely susceptible to electromyographic (EMG) contamination from jaw and neck muscles.	Adjunctive assessment
Neurodegenerative diseases (e.g., Alzheimer’s)	Global slowing of Peak Alpha Frequency (PAF) (< 8 Hz); increased Theta/Delta power temporally; reduced posterior coherence.	Moderate to high	Findings can be confounded by physiological aging, vascular white matter lesions, and polypharmacy.	Adjunctive assessment/Investigational for large-scale screening
Epilepsy (and pharmaco-EEG)	Interictal background slowing; altered phase coherence; early predictive network changes following pharmacotherapy.	Low to moderate	Volume conduction limits spatial resolution for precise focal localization compared to invasive EEG or structural imaging.	Conventional EEG for clinical diagnosis/QEEG for adjunctive monitoring
Chronic neuropathic pain	Thalamocortical Dysrhythmia (TCD): increased Theta activity pathologically coupled with high-frequency Gamma oscillations.	Moderate	Findings are frequently masked by centrally acting analgesics (e.g., opioids, gabapentinoids, antidepressants).	Research use only
Post-COVID-19 syndrome	Generalized cortical slowing (Theta/Alpha increase); asymmetrical SMR distribution; increased Beta2/SMR ratio.	Low (emerging)	Lack of large-scale, controlled studies; electrophysiological patterns are highly non-specific and overlap with chronic fatigue.	Research use only

^1^Levels of evidence were assigned using a structured narrative framework based on: (1) the highest available study design supporting a given application (guidelines, meta-analyses, systematic reviews, randomized or prospective studies, versus small retrospective or exploratory studies), (2) replication across independent cohorts, (3) evidence of diagnostic or predictive performance beyond a single center, (4) current clinical or regulatory readiness, and (5) the severity of methodological limitations such as heterogeneity, artifact susceptibility, inadequate standardization, and lack of longitudinal validation.

### 4.1 Neurodevelopmental disorders: ADHD and autism spectrum

Currently, the clinical utility of QEEG is extensively investigated in the diagnosis of neurodevelopmental disorders (Adamou et al., 2020). In the case of ADHD, spectral analysis has shown that the disorder is associated with specific neurobiological patterns that go beyond behavioral descriptions alone (Loo and Makeig, 2012). A significant biomarker supporting the diagnostic process, is the TBR, which has been cleared by the U. S. Food and Drug Administration (FDA) strictly as an adjunctive diagnostic tool rather than a standalone diagnostic criterion (Gloss et al., 2016; Adamou et al., 2020). In a substantial portion of children with ADHD, increased power in the Theta band is observed in frontal leads, accompanied by a decrease in spectral density in the Beta band (Bresnahan and Barry, 2002; Loo and Makeig, 2012). This pattern positively correlates with the clinical severity of attention deficits and impulsivity (Loo and Makeig, 2012; McVoy et al., 2019b; Wang et al., 2023).

However, electrophysiological profiles in ADHD exhibit significant developmental variability (Loo and Makeig, 2012; Clarke et al., 2019). In adult patients, particularly high-functioning individuals who have developed compensatory strategies over time, the topographic image often changes (Bresnahan and Barry, 2002; Clarke et al., 2019). Instead of generalized frontal slowing, increased activity in the Beta band is frequently recorded during a QEEG examination (Bresnahan and Barry, 2002; Clarke et al., 2019). This pattern is an electrophysiological correlate of heightened cognitive effort and co-occurring anxiety tension, which can effectively mask primary attention deficits in standard psychometric testing. QEEG may facilitate the objective verification of these hidden aberrations, which can be valuable for proper differential diagnosis (Bresnahan and Barry, 2002).

In the diagnosis of ASD, QEEG analysis provides detailed data on the organization of cortical networks (connectivity) (Coben et al., 2008; O’Reilly et al., 2017; Geng et al., 2023). A frequently observed electrophysiological phenotype in populations of children with mild ASD symptoms is the coexistence of hypocoherence for long-distance connections (indicating weakened integration between distant brain regions) and hypercoherence for local connections (reflecting excessive coupling of adjacent neuronal circuits) (Barttfeld et al., 2011; Milovanovic and Grujicic, 2021; Garcés et al., 2022; Geng et al., 2023). Such functional architecture constitutes the neurobiological basis for difficulties in sensory integration and cognitive flexibility (Coben et al., 2008; O’Reilly et al., 2017).

Furthermore, preliminary evidence suggests that the precise identification of individual network aberrations in the resting state with closed eyes determines the effectiveness of neuromodulatory therapies (Murias et al., 2007; Coben et al., 2008). Personalizing Neurofeedback training protocols based on the baseline QEEG recording leads to an objective and measurable reduction in attention deficits co-occurring with ASD (Coben et al., 2008). However, as some of these specific neuromodulatory findings are currently derived from a limited number of closely related research cohorts, independent large-scale replication is required to validate their broad clinical applicability.

### 4.2 Mood and anxiety disorders: affective biomarkers

In the clinical practice of adult psychiatry, QEEG spectral analysis is evolving into an objective tool supporting the differential diagnosis of mood and anxiety disorders, facilitating, among other things, the differentiation of unipolar depression from episodes in the course of bipolar affective disorder. Reviews and meta-analyses indicate FAA as one of the most thoroughly researched electrophysiological markers of depression. Left-sided increased Alpha power (higher Alpha in F3/F7/Fp1 than in F4/F8/Fp2) corresponds to a relative hypoactivation of the left frontal cortex and is associated with negative affect and withdrawal behaviors (Dharmadhikari et al., 2019; Fitzgerald, 2024; Luo et al., 2025). At the same time, newer studies show that the effect has a small magnitude and limited diagnostic value alongside significant outcome heterogeneity, suggesting that FAA should be treated as an auxiliary rather than a standalone biomarker (Dharmadhikari et al., 2019; Kołodziej et al., 2021; Luo et al., 2025).

Conversely, in the presentation of anxiety disorders, the dominant QEEG phenotype is localized or generalized hyperactivity in the fast frequency band (the so-called high Beta or Hi-Beta, typically in the 20–30 Hz range). This pattern represents a direct electrophysiological manifestation of chronic cortical hyperarousal and the impairment of inhibitory mechanisms that condition the state of relaxation (Díaz et al., 2019; Lin et al., 2021; Boby et al., 2024; Wang et al., 2025). A relationship between a higher TBR and anxiety severity has also been demonstrated (Byeon et al., 2023). It should be further emphasized that objectifying these hidden dysfunctions using multicolored topographic maps has fundamental psychoeducational significance in the treatment process. The spatial visualization of neurophysiological markers helps patients externalize their symptoms and rationalize subjective discomfort, effectively reducing the level of self-stigmatization and significantly optimizing engagement indicators in the therapeutic process (compliance).

### 4.3 Neurology: epilepsy and neurodegenerative processes

The application of QEEG in neurology extends beyond the traditional detection of paroxysmal discharges. In epilepsy, particularly in drug-resistant cases, quantitative analysis allows for the assessment of bioelectric background activity during interictal periods. Changes in band power and coherence can predict the effectiveness of pharmacological treatment, including innovative therapies using CBD. Monitoring changes in QEEG under the influence of CBD may provide adjunctive indicators of the neuroprotective and stabilizing effects of this substance, even before full clinical remission of seizures occurs (Kopańska et al., 2025d). Furthermore, QEEG examination in patients enrolled in an expanded access program revealed that background parameters [e.g., Beta power, the aperiodic component of the EEG power spectrum (1/f slope), correlations, and coherence] differed between responders and non-responders already in the baseline recording, and a model combining coherence, correlation, and sex predicted the response to CBD with an accuracy of approximately 74% (Armstrong et al., 2022). QEEG is also applied in evaluating the effectiveness of other antiepileptic drugs and monitoring network changes under the influence of pharmacotherapy (Fonseca et al., 2022; Assenza et al., 2024; Wagh et al., 2025).

Beyond the assessment of background activity and general pharmacological response, QEEG serves critical functions in the spatial localization of epileptic foci and the dynamic prediction of seizure events (Nemtsas et al., 2017; van Mierlo et al., 2020; Singh et al., 2022). Advanced source imaging techniques, combined with the quantitative analysis of interictal epileptiform discharges (IEDs), such as calculating spike frequency, amplitude mapping, and spatial propagation patterns, provide precise topographical data that is increasingly utilized in pre-surgical evaluation (Sharma et al., 2018; Avigdor et al., 2024; Vorderwülbecke et al., 2025). Furthermore, modern QEEG analytics actively leverage non-linear dynamics and EEG complexity measures, such as entropy and fractal dimension, as predictive biomarkers (Lau et al., 2022; Abhishek et al., 2024; Porcaro et al., 2024). The transition from the interictal to the preictal state is frequently characterized by a detectable, pathological reduction in EEG complexity, allowing for the timely prediction of impending seizures (Zhang et al., 2020; Abhishek et al., 2024). In the realm of continuous pharmacotherapy, these quantitative metrics are invaluable not only for predicting the overarching response to treatments but also for the meticulous adjustment and optimization of standard antiepileptic drug (AED) dosages (Höller et al., 2018; Reynolds et al., 2023; Assenza et al., 2024). By monitoring drug-induced temporal changes in spectral power and functional connectivity, clinicians can objectively titrate AEDs to maximize seizure control while simultaneously minimizing neurotoxic side effects and cognitive blunting, thereby establishing a robust, objective framework for personalized epilepsy management (Lanzone et al., 2021; Assenza et al., 2024; Bonacci et al., 2024).

Simultaneously, a dynamically developing area of quantitative neurodiagnostics is the early detection of neurodegenerative diseases and pathological Aging-Associated Cognitive Decline (AACD). Although physiological aging of the central nervous system involves a natural, mild slowing of the Peak Alpha Frequency (PAF), its rapid degradation acquires highly pathological significance. A reduction in the PAF parameter, co-occurring with a pathological increase in the spectral density of slow Theta and Delta bands in the topographic projection of the temporal lobes, is increasingly recognized as an early electrophysiological marker of Alzheimer’s disease (AD). Crucially, to validate its clinical utility, these functional aberrations must be correlated with established, gold-standard pathophysiological biomarkers, such as cerebrospinal fluid (CSF) levels of amyloid-beta (Aβ) and phosphorylated tau, as well as molecular Positron Emission Tomography (PET) imaging. While QEEG is highly sensitive to progressive cortical pathway disconnections, often detecting functional decline before macroscopic nerve tissue atrophy becomes visible on standard structural MRI, it is essential to clarify its diagnostic scope. QEEG is exceptionally well-suited as a non-invasive, cost-effective tool for large-scale cognitive screening and adjunctive functional assessment, rather than for the definitive diagnosis of AD (Musaeus et al., 2018; Popa et al., 2020; Yao et al., 2022; Yuan and Zhao, 2025).

### 4.4 New fields: pain medicine and pharmaco-EEG

QEEG also opens new diagnostic perspectives in fields previously weakly correlated with clinical neurophysiology, such as the diagnosis of chronic pain or the impact of infections on the central nervous system.

The objectification of pain remains one of the most formidable challenges in clinical medicine, but QEEG offers promising insights into distinguishing varying pain phenotypes (Mussigmann et al., 2022; Zis et al., 2022; Adebisi et al., 2025). In chronic neuropathic pain, the dominant electrophysiological signature is Thalamocortical Dysrhythmia (TCD), which typically manifests as an abnormal increase in resting-state Theta power that is pathologically coupled with high-frequency Gamma oscillations, predominantly over the somatosensory cortex (Llinás et al., 1999; Stern et al., 2006; Walton et al., 2010). Conversely, acute nociceptive pain often presents with distinct dynamic changes, primarily characterized by widespread Alpha desynchronization and a proportional increase in Beta activity, reflecting acute cortical arousal and active nociceptive processing (Modares-Haghighi et al., 2021; Ahmad and Barkana, 2025; Kannan et al., 2025). In cases of psychogenic or functional pain, the primary somatosensory aberrations characteristic of TCD are typically absent. Instead, the QEEG profile frequently mirrors affective dysregulation, displaying markers such as FAA or excessive frontal high-Beta activity associated with emotional distress and somatic anxiety (Ryu et al., 2024; Kannan et al., 2025).

While the diagnostic value of QEEG in pain medicine lies in its potential to objectify the patient’s functional experience and monitor the neuroplastic changes induced by neuromodulatory interventions, its limitations must be critically acknowledged (Dos Santos Pinheiro et al., 2016; Ta Dinh et al., 2019; Mussigmann et al., 2022; Zis et al., 2022; Zebhauser et al., 2023). Pain is inherently a multidimensional subjective experience, and its electrophysiological signatures frequently overlap with those of comorbid depression or anxiety (Vanneste et al., 2018; Mussigmann et al., 2022; Zebhauser et al., 2023). Furthermore, the objective assessment of pain via QEEG is heavily confounded by the profound spectral alterations induced by centrally acting analgesics, such as opioids, gabapentinoids, and antidepressants, which can independently drive widespread cortical slowing or shift peak frequencies, thereby potentially masking the primary pain-related biomarkers (Dos Santos Pinheiro et al., 2016; Mussigmann et al., 2022; Zebhauser et al., 2023). Recent preliminary literature also indicates the potential utility of QEEG in evaluating the so-called “brain fog” following COVID-19 (Morga et al., 2023; Góral-Półrola et al., 2024). In patients with chronic fatigue and memory deficits, QEEG reveals a pathological increase in power in the Theta and Alpha bands, correlating with generalized cortical slowing. At the same time, the lateralization of the SMR rhythm has been recorded via dominance in the right C4 lead relative to the left C3 alongside a bilateral increase in the ratio of fast Beta2 waves to SMR (Kopańska et al., 2026). This profile constitutes a sensitive electrophysiological biomarker of the coexistence of attention deficits and compensatory cortical hyperarousal, optimizing the monitoring of post-infectious syndromes and neurorehabilitation processes. Importantly, preliminary reports suggest that the application of targeted neuromodulatory training based on a precise QEEG diagnosis in patients with post-COVID syndrome may support the reduction of these deficits and potentially improve cognitive functions (Pąchalska and Góral-Półrola, 2022). It must be explicitly stated that evidence regarding QEEG in post-COVID-19 syndrome relies heavily on specific research groups, and independent replication remains currently limited.

In psychiatry, the field of pharmaco-EEG is developing. Specific QEEG profiles can indicate a patient’s potential response to certain groups of psychotropic drugs, such as SSRI vs. dopaminergic medications, which helps avoid “trial-and-error” strategies when selecting pharmacotherapy (Widge et al., 2019; Huidobro et al., 2025). Furthermore, the high sensitivity of spectral analysis allows for the precise objectification not only of strictly pharmacological interventions but also of the impact of targeted nutritional strategies and supplementation on the brain’s bioelectrical activity. While emerging, preliminary reports suggest that the optimization of nutritional status may act as a cofactor modulating network parameters, independent replication remains necessary to confirm its influence on the ultimate efficacy of neuropsychiatric therapies (Kopańska et al., 2025a).

The clinical significance of QEEG lies not only in supporting diagnosis but primarily in providing guidance for therapeutic interventions and personalized treatment. QEEG analysis enables the design of individual Neurofeedback or neuromodulation protocols (rTMS/tDCS), the precise determination of dysfunction localization and its resonance frequency (IAF), as well as monitoring the effects of both pharmacological and behavioral therapies (Fingelkurts and Fingelkurts, 2022). QEEG also makes it possible to objectify “invisible” cognitive disorders in high-functioning individuals or those masking deficits and to differentiate overlapping clinical presentations, for example, ADHD vs. anxiety disorders or depressive pseudodementia vs. dementia (Chabot et al., 2005).

## 5 Clinical significance and therapeutic implications

The clinical significance of QEEG continues to evolve. Based on the analysis of accumulated evidence, this method serves as a highly valuable adjunctive, functional, and hypothesis-generating tool in modern neurodiagnostics. The transition from subjective behavioral assessment to objective quantification of neurophysiological processes represents a significant paradigm shift that medicine has awaited for decades. QEEG bridges the gap between static structural imaging and the dynamic nature of psychological phenomena, offering insights into the functional organization of the brain with millisecond precision (Kopańska et al., 2024). In the face of the heterogeneity of psychiatric and neurological disorders, QEEG provides promising adjunctive biomarkers for therapeutic interventions, facilitating the transition from treatment based on general protocols to personalized medicine.

### 5.1 Differential diagnosis and decomposition of complex clinical presentations

A key area where QEEG demonstrates its clinical value is differentiating overlapping symptoms (the so-called overlap phenomenon) and unmasking compensatory mechanisms, allowing clinicians to move beyond simplified diagnostic schemes (Yadollahpour and Nasrollahi, 2016; Fingelkurts and Fingelkurts, 2022; Yao et al., 2022). In clinical practice, patients are frequently encountered who report subjective cognitive difficulties despite normal neuropsychological test results. This situation particularly applies to high-functioning adults with ADHD, whose developed coping strategies mask primary deficits. QEEG analysis may help to unmask these hidden neuronal costs. For example, in high-functioning individuals with ADHD, instead of the classic excess of slow Theta waves, paradoxical hyperactivity of Beta waves is recorded, indicating chronic overload and anxiety (Bong and Kim, 2021; Ciftci and Alp, 2025; Ölçüoğlu, 2025). Identifying this pattern may help protect patients from misdiagnosis involving anxiety or personality disorders and the implementation of incorrect pharmacotherapy (Chabot et al., 2005). It is also worth emphasizing that baseline electrophysiological parameters and susceptibility to neuromodulation are significantly influenced by diverse sociodemographic and environmental factors. Recent evidence indicates that dietary habits, nutritional status (including supplementation), and general public health indicators strongly modulate the brain’s bioelectrical activity, making QEEG a useful tool in assessing the impact of lifestyle on the functioning of neuronal networks (Hejda et al., 2025; Łucka et al., 2025).

QEEG also provides critical biomarkers in other diagnostic dilemmas. It may help distinguish patients with early-stage dementia, who exhibit global brain slowing and a drop in PAF below 8 Hz, from patients with severe depression, where the QEEG recording is typically normal or shows only FAA (Livinț Popa et al., 2021; Simfukwe et al., 2025b; Yuan and Zhao, 2025). Furthermore, this method enables the differentiation of behaviors in children, including separating symptoms resulting from early childhood trauma (manifesting as specific dysfunctions in the temporal lobes and limbic system known as temporal hot spots) from typical frontal dysfunctions observed in ADHD (Chabot et al., 2005; McVoy et al., 2019b; Bong and Kim, 2021). In ASDs, the assessment of functional connectivity patterns in the resting state becomes the starting point for understanding the patient’s individual sensory profile, which is difficult to achieve solely through observation (McVoy et al., 2019b; Ferreira et al., 2025; Tenev et al., 2025).

### 5.2 Advances in therapeutics: QEEG, Neurofeedback, and neuromodulation—modern perspectives

Modern neurotherapy increasingly utilizes advanced methods of QEEG, Neurofeedback and broadly defined neuromodulation. Neurofeedback is a technique of endogenous neuromodulation that enables the conscious regulation of selected brain activity parameters through real-time feedback. In practice, it involves recording the EEG signal (or fMRI), processing it, and presenting visual or auditory feedback to the patient that reflects the current state of specific brain waves or neuronal activity patterns (Enriquez-Geppert et al., 2017; Sitaram et al., 2017; Batail et al., 2019; Sulzer et al., 2024; Tosti et al., 2024). The participant learns how to modify their brain activity through instrumental conditioning to achieve a desired effect, such as increasing Alpha wave power or decreasing the TBR (Sitaram et al., 2017; Loriette et al., 2021; Rubia et al., 2021; Tosti et al., 2024). Furthermore, the progressive integration of QEEG analysis with novel neurotechnologies opens unprecedented opportunities for precise mapping and targeted modulation of cortical plasticity, thereby significantly optimizing neurorehabilitation processes (Kropotov et al., 2014).

In evaluating the clinical efficacy of Neurofeedback, it is crucial to distinguish between various standardized training protocols and their specific target populations (Enriquez-Geppert et al., 2019; Glaubig et al., 2026). The training protocol is selected individually based on QEEG and may include various frequency bands (Alpha, Beta, Theta), electrode locations, or feedback types (Marzbani et al., 2016; Enriquez-Geppert et al., 2017; Tosti et al., 2024). For instance, Sensorimotor Rhythm (SMR) training (12–15 Hz) typically targets the sensorimotor cortex and is primarily utilized to enhance inhibitory motor control and reduce hyperactivity in ADHD, as well as to stabilize neural networks in epilepsy (Enriquez-Geppert et al., 2019; Morales-Quezada et al., 2019; Dousset et al., 2024). Conversely, TBR training focuses on the frontal and central regions specifically to alleviate symptoms of inattention and cognitive underarousal (Bluschke et al., 2016; Enriquez-Geppert et al., 2019). Meanwhile, the training of Slow Cortical Potentials (SCPs), which involves the conscious regulation of overall cortical excitability through phasic electrical shifts, has demonstrated distinct efficacy in managing impulsivity and epileptic seizures (Enriquez-Geppert et al., 2019; Morales-Quezada et al., 2019).

However, the therapeutic success of these standardized protocols is highly contingent upon the patient’s baseline neurophysiological characteristics (Slater et al., 2022; Boxum et al., 2025; Ölçüoğlu, 2025). Because a single clinical diagnosis, such as ADHD, can manifest through highly heterogeneous electrophysiological phenotypes (e.g., classical excess Theta versus compensatory high-Beta), applying a standardized protocol may result in treatment failure if the patient’s baseline EEG does not align with the assumed trained parameters (Byeon et al., 2020; Ölçüoğlu, 2025). Consequently, there is a growing consensus that individualized Neurofeedback protocols, precisely tailored to correct the specific dysregulations identified in the patient’s baseline QEEG, theoretically offer superior therapeutic targeting (Loo and Makeig, 2012; Boxum et al., 2025; Ölçüoğlu, 2025). While preliminary clinical evidence suggests that QEEG-guided interventions may yield better clinical outcomes by addressing unique network aberrations rather than generic behavioral symptoms, definitive confirmation of their clinical superiority over standardized protocols still awaits large-scale, head-to-head RCTs (Loo and Makeig, 2012; Ölçüoğlu, 2025).

Neurofeedback finds numerous applications in clinical practice, including the therapy of ADHD, anxiety and depressive disorders, PTSD, addictions, epilepsy, insomnia, and autism, as well as serving as an adjunctive treatment in pain management, post-stroke rehabilitation, or the improvement of cognitive functions in the elderly (Niv, 2013; Marzbani et al., 2016; Enriquez-Geppert et al., 2017; Markiewicz, 2017; Patel et al., 2020; Loriette et al., 2021; Rubia et al., 2021). Promising results are observed in the area of neurorehabilitation for patients following traumatic brain injuries (TBI). Preliminary studies suggest that individualized Neurofeedback protocols may help alleviate chronic sleep disorders and lower the level of generalized anxiety and symptoms of post-traumatic stress disorder (PTSD), which can support the successful social and professional reintegration of these patients (Pąchalska et al., 2023). It is also increasingly used to optimize cognitive and emotional performance in athletes and pilots (Corrado et al., 2024; Alexiou, 2025).

Research suggests the potential effectiveness of Neurofeedback in improving attention and working memory and in reducing ADHD or anxiety symptoms (Rubia et al., 2021; Fernández et al., 2024; Ölçüoğlu, 2025). Meta-analyses have shown moderate therapeutic effects for ADHD and depression, especially when using standard protocols (Fernández-Alvarez et al., 2022; Westwood et al., 2025). In the case of chronic pain, a significant reduction in pain intensity of up to 30–80% is observed in selected patient groups (Patel et al., 2020; Mussigmann et al., 2025). Initial studies suggest that targeted, QEEG-based neuromodulatory protocols may be safely applied in the geriatric population, potentially contributing to the alleviation of chronic neuropathic pain and improvement in the quality of life of older adults (Chmiel et al., 2026). Despite its clinical implementation, the definitive efficacy of Neurofeedback remains a subject of intense scientific debate, primarily due to the profound influence of the placebo effect (Loriette et al., 2021; Fernández-Alvarez et al., 2022; Kalokairinou et al., 2022). Critics rightly argue that the clinical improvements observed in Neurofeedback trials may often be driven by non-specific treatment factors rather than the targeted operant conditioning of specific brainwaves (Thibault and Raz, 2017; Kalokairinou et al., 2022). These non-specific variables encompass the therapeutic alliance, the generalized cognitive effort and attention training required during the task, and the patient’s inherent motivation and expectation of clinical improvement (Thibault and Raz, 2017; Kadosh and Staunton, 2019; Schönenberg et al., 2021a).

To rigorously isolate the specific neurobiological effects of Neurofeedback from these confounding psychosocial variables, study designs must incorporate sham-controlled groups (Arnold et al., 2013a, 2013b; Aggensteiner, 2021). In such paradigms, control participants receive simulated visual or auditory feedback that is disconnected from their actual real-time EEG activity (Arnold et al., 2013a, 2021; Kalokairinou et al., 2022). However, executing true double-blind RCTs in Neurofeedback presents formidable methodological challenges (Arnold et al., 2013a; Kalokairinou et al., 2022; Lam et al., 2022). Effective blinding is notoriously difficult. Perceptive patients may recognize a lack of contingent control over the sham feedback, while therapists administering the protocol may inadvertently introduce bias if they become unblinded (Arnold et al., 2013a; Schönenberg et al., 2017). Consequently, claims regarding the efficacy of Neurofeedback must be interpreted with caution (Schönenberg et al., 2017; Arnold et al., 2021; Rice et al., 2024). To firmly establish Neurofeedback as a robust, evidence-based clinical therapy rather than a product of placebo mechanisms and statistical inconsistencies found in small-sample studies, future research must mandate rigorous, sham-controlled methodologies (Arnold et al., 2013b; Loriette et al., 2021; Zhao et al., 2026). Detailed advantages and disadvantages of this method have been compiled in Table 4.

**Table 4:** Summary of advantages, limitations, and challenges of Neurofeedback/EEG-Biofeedback therapy (Batail et al., 2019; Schönenberg et al., 2021b; Chikhi et al., 2024; Molaeizadeh et al., 2025).

Advantages	Limitations and challenges
No risk of serious adverse effects, non-invasive nature	Time-consuming, multiple sessions are required (20–40)
Ability to customize the protocol to the individual’s QEEG profile	High costs of equipment and specialist labor
Durability of effects (changes can persist for months after therapy ends)	Lack of protocol standardization, high variability between studies
Multidimensional impact on cognitive, emotional, and behavioral functions	Placebo effect, difficulties distinguishing specific from non-specific effects
Possibility of integration with other methods, e.g., CBT or mindfulness	Limited availability of high-quality RCT studies
	Variable patient motivation, effectiveness depends on participant engagement

Beyond endogenous neuromodulation via Neurofeedback, the therapeutic landscape is increasingly driven by exogenous non-invasive brain stimulation (NIBS) techniques, predominantly repetitive transcranial magnetic stimulation (rTMS) and transcranial direct current stimulation (tDCS) (Padberg et al., 2021; Hyde et al., 2022; Kesikburun, 2022). Unlike Neurofeedback, these interventions are supported by an ample body of rigorous, sham-controlled clinical evidence and convincing mechanistic explanations, leading to their established efficacy in treating several psychiatric conditions, notably treatment-resistant depression (Hyde et al., 2022). In this context, QEEG is emerging as a critical navigational and predictive tool (Desarkar et al., 2024; Yousefian et al., 2025). Rather than relying exclusively on standardized anatomical landmarks (e.g., targeting the F3 coordinate for the left dorsolateral prefrontal cortex), QEEG-guided NIBS utilizes individual functional mapping to optimize target selection based on the patient’s unique topographical dysregulations (Padberg et al., 2021; Desarkar et al., 2024). Furthermore, quantitative spectral analysis allows for the precise calibration of stimulation parameters; for instance, adjusting rTMS protocols to the patient’s Individual Alpha Frequency (IAF) can significantly enhance intrinsic network entrainment and therapeutic resonance (Padberg et al., 2021; Baradits et al., 2026). Finally, baseline QEEG parameters, such as pre-treatment functional connectivity metrics and specific power ratios, are actively investigated as robust predictive biomarkers (Watts et al., 2022; Zhu et al., 2024). These indicators help estimate treatment efficacy and stratify patients who are most likely to respond positively to specific rTMS or tDCS protocols, thereby establishing a more objective framework for personalized neuromodulation (Karimi et al., 2025; Yousefian et al., 2025).

### 5.3 Psychoeducational aspect and patient compliance

An increasingly recognized, yet historically underappreciated, aspect of QEEG application is its potential psychoeducational impact (Benedetti et al., 2023; Collura et al., 2025). Providing patients with a visual representation of their electrophysiological brain activity, often in the form of intuitive, color-coded topographic maps, serves as a pivotal catalyst in the therapeutic process (Nuwer, 1988; Benedetti et al., 2023; Vafaei et al., 2023). This visual feedback facilitates the crucial externalization of psychiatric and neurodevelopmental symptoms (Vafaei et al., 2023; Collura et al., 2025; Liew et al., 2025). By shifting the patient’s internal narrative from subjective character flaws (e.g., the belief that one is simply “lazy” or “unmotivated”) to objective, quantifiable biological dysregulations (e.g., observing a specific difficulty with cortical inhibition or frontal lobe underarousal), QEEG helps demystify the disorder (de Oliveira and Dias, 2023; Sterzer et al., 2025; Blum et al., 2026).

The clinical value of this neurobiological objectification extends beyond simple symptom explanation. It may help mitigate the internalized stigma and guilt that frequently act as primary barriers to successful psychiatric treatment (de Oliveira and Dias, 2023; Blum et al., 2026). By enhancing the patient’s health literacy through tangible brain mapping, clinicians can foster a much stronger therapeutic alliance (de Oliveira and Dias, 2023; Tessier et al., 2023). This approach effectively transitions the patient from a passive recipient of psychiatric care to an active, informed participant (Danzl et al., 2012; de Oliveira and Dias, 2023; Blum et al., 2026). Consequently, this advanced form of visually supported psychoeducation may positively influence treatment adherence (compliance) (de Oliveira and Dias, 2023; Biswal et al., 2024; Baabouchi et al., 2025). Clinical experience indicates that patients are considerably more likely to engage with and commit to demanding therapeutic interventions, such as prolonged Neurofeedback protocols or repetitive neuromodulation, when they possess a clear, objective understanding of both their baseline physiological dysregulations and the specific neuroplastic goals of the therapy (Danzl et al., 2012; Loriette et al., 2021; Green et al., 2025).

However, it is important to emphasize that enhanced patient compliance currently remains a plausible and highly promising clinical benefit that requires further rigorous empirical validation, rather than an established direct effect of QEEG implementation.

### 5.4 Practical interpretative framework and common pitfalls

To effectively translate QEEG data into clinical practice, interpretation should follow a cohesive, sequential framework rather than relying on isolated metrics (Babiloni et al., 2020; Collura et al., 2025). The initial and arguably most critical stage is rigorous data quality control, which involves the strict visual and algorithmic verification of the raw trace to identify and exclude non-cerebral artifacts (Duffy et al., 1994; Li et al., 2022; Collura et al., 2025). Once a clean signal is established, the analysis proceeds to spectral evaluation and topographic mapping, allowing for the assessment of absolute and relative power distributions across standard frequency bands (Jeong et al., 2022; Collura et al., 2025; Liew et al., 2025). This is subsequently complemented by a functional connectivity assessment, where coherence and phase relationships are evaluated to gage broader network integrity (Murugappan, 2022; Yuan and Zhao, 2025; Prichep et al., 2026). Crucially, these raw and relational metrics must then be compared against age- and sex-matched normative databases, transforming the data into Z-scores to objectively quantify statistical deviance (Duffy et al., 1994; Ko et al., 2021; Jeong et al., 2022). However, the culmination of this process is the clinical correlation. QEEG findings cannot be interpreted in a vacuum. They must be carefully contextualized within the patient’s specific behavioral symptoms, medical history, and ongoing pharmacotherapy (Duffy et al., 1994; Pinheiro et al., 2026).

Alongside this structured approach, clinicians must remain vigilant regarding common interpretative pitfalls that can easily confound diagnosis (Collura, 2020; Pinheiro et al., 2026). Frequent errors include the misinterpretation of generalized slow-wave activity, which may simply reflect transient patient drowsiness rather than true cortical pathology (Jeong et al., 2022; Leach et al., 2023). Similarly, clinicians must be cautious of false elevations in high-frequency Beta or Gamma power, which are frequently driven by unrecognized electromyographic (EMG) artifacts stemming from subtle jaw or neck tension (Grosselin et al., 2019; Jeong et al., 2022; Pinheiro et al., 2026). Moreover, severe distortion of topographic amplitude maps can occur if an inappropriate reference electrode scheme is applied without recognizing its spatial limitations (Babiloni et al., 2020; Jeong et al., 2022; Liew et al., 2025). Finally, it is essential to recognize that interpretative priorities must dynamically adapt to the specific clinical scenario (Babiloni et al., 2020; Ko et al., 2021; Murugappan, 2022). For instance, while the detection of global slowing and a reduced Peak Alpha Frequency (PAF) is paramount in neurodegenerative screening, the identification of localized Beta excess and specific connectivity aberrations takes definitive precedence in the evaluation of adult ADHD or anxiety disorders (Smailovic and Jelic, 2019; Kesebir et al., 2022; Simfukwe et al., 2025b; Prichep et al., 2026).

## 6 Future directions and technological integration

The significance of QEEG is expected to grow alongside the development of artificial intelligence (AI) and machine learning algorithms (Yao et al., 2022; Bomatter et al., 2024). Integrating extensive normative databases with Big Data systems is already paving the way for the creation of automated diagnostic classifiers with high sensitivity and specificity (Tveit et al., 2023; Li et al., 2025). It is predicted that brain mapping will become a standard screening procedure in psychiatric and neuropsychological clinics, analogous to routine ECG in cardiology (Keikhosrokiani et al., 2024; Baydili et al., 2025). However, the clinical integration of AI faces critical bottlenecks, including the “black-box” nature and poor interpretability of complex models, distribution bias in training datasets, and a high risk of overfitting in small-sample studies (Chen et al., 2023; Cross et al., 2024; Frasca et al., 2024). Another promising direction is the miniaturization of devices into wearable technology, enabling long-term monitoring outside the laboratory (Uchitel et al., 2021; Gao et al., 2022). Yet, this field must first overcome significant practical hurdles, such as insufficient signal-to-noise ratios, mismatches with standardized clinical normative databases, and a lack of clinical-grade quality control (Keikhosrokiani et al., 2024; Olatinwo and Orji, 2026). This increases the method’s accessibility for a broad patient population (Yao et al., 2022; Tveit et al., 2023; Keikhosrokiani et al., 2024; Amico and Koberda, 2025). Ultimately, the most profound barrier to the global clinical translation of QEEG remains the lack of universally accepted, uniform clinical interpretation standards (Norori et al., 2021; Chen et al., 2023; Rutkowski and Saab, 2025).

## 7 Summary and conclusion

The integration of QEEG into clinical and research settings represents a notable advancement in modern neurodiagnostics. As demonstrated in this review, QEEG extends beyond the qualitative visual inspection of brain electrical activity, serving as a translational tool that seeks to correlate subjectively reported clinical symptoms with measurable neurophysiological indicators.

A primary clinical interest in QEEG lies in its potential to inform the therapeutic process. By identifying distinct electrophysiological profiles, such as compensatory high-Beta activity in adult ADHD, early spectral shifts in neurodegeneration, or overlapping affective markers, QEEG provides supplementary data that may help clinicians refine diagnostic assessments. Consequently, this method supports the broader transition toward personalized medicine. It offers an exploratory framework where neuromodulatory protocols (such as Neurofeedback, tDCS, and rTMS) and pharmacological treatments might be tailored and monitored based on objective functional metrics rather than solely on behavioral criteria.

In summary, QEEG should be regarded not as a standalone diagnostic transformation, but as a valuable adjunctive tool. While its capacity to optimize treatment trajectories, such as potentially reducing time to remission and mitigating polypharmacy, represents a compelling hypothesis, these clinical benefits require rigorous validation through prospective, sham-controlled, and large-scale randomized trials. As digital analytics and artificial intelligence continue to advance, QEEG shows considerable promise. Once critical standardization and methodological challenges are overcome, it has the potential to significantly support the personalization of therapy and contribute to the future of evidence-based precision neuropsychiatric care.
